# Antimicrobial Activity and Proposed Action Mechanism of Linalool Against *Pseudomonas fluorescens*

**DOI:** 10.3389/fmicb.2021.562094

**Published:** 2021-01-28

**Authors:** Fengyu Guo, Qianping Chen, Qiong Liang, Ming Zhang, Wenxue Chen, Haiming Chen, Yonghuan Yun, Qiuping Zhong, Weijun Chen

**Affiliations:** College of Food Science and Technology, Hainan University, Haikou, China

**Keywords:** linalool, antibacterial mechanism, *Pseudomonas fluorescens*, membrane damage, metabolic respiration, TCA cycle

## Abstract

In this study, linalool, one of the principal components of essential oils, was used as an antibacterial agent to investigate the antibacterial activity and mechanism of linalool against *Pseudomonas fluorescens.* The reduction in membrane potential (MP), leakage of alkaline phosphatase (AKP) and the release of macromolecules, including DNA, RNA and protein confirmed that damage to cell wall membrane structure and leakage of cytoplasmic contents were due to the linalool treatment. Furthermore, the decrease of enzyme activity, including the succinate dehydrogenase (SDH), malate dehydrogenase (MDH), pyruvate kinase (PK), and ATPase indicated that linalool could lead to metabolic dysfunction and inhibit energy synthesis. In addition, the activity of respiratory chain dehydrogenase and metabolic activity of respiration indicated that linalool inhibits cellular respiration. These results revealed that linalool had strong antibacterial activity against *P. fluorescens* via membrane damage, bacterial metabolic and oxidative respiratory perturbations, interfering in cellular functions and even causing cell death. It was suggested that linalool may be a new potential source as food antiseptics in food systems.

## Introduction

In recent years, aromatic plant extracts have been widely used in the field of food preservation (Brenes and Roura, 2010; Prakash et al., 2015). Essential oils (EOs) are naturally occurring antimicrobial agents found in many plants and are the main reason for the antimicrobial properties of aromatic plant extracts (Brenes and Roura, 2010; Callaway et al., 2011). At present, many of the most widely used food preservatives are synthetic products. However, synthetic products have many disadvantages, such as potential carcinogenicity, teratogenicity, environmental pollution, and acute toxicity (Faleiro, 2011). Therefore, people are eager to find natural food additives with which to replace the synthetic chemical additives (Fratianni et al., 2010). EOs are an ideal choice because of their significant antibacterial properties and safety (Calo et al., 2015).

Linalool (C_1__0_H_1__8_O), also known as 3,7-dimethyl-1,6-octadien-3-ol, is a monoterpene alcohol, which is found in the EOs extracted from more than 200 plants worldwide, such as Thymus vulgaris (thyme) EOs and Juniperus communis (juniper) EOs (Aprotosoaie et al., 2014; Pereira et al., 2018). This monoterpene alcohol poses antioxidant, anti-inflammatory, and anticancer activities (Herman et al., 2016) and exhibits antibacterial activity against *Staphylococcus aureus* NCTC 10788, *Pseudomonas aeruginosa* NCTC 12924, and *Escherichia coli* NCTC 12923 (Herman et al., 2016). Consequently, it is widely used in pharmaceuticals, cosmetics and food additives (Calo et al., 2015). Annually, approximately 1,000 metric tons of linalool are consumed globally each year (Aprotosoaie et al., 2014). In this case, it is very promising to develop natural antimicrobials from linalool.

*Pseudomonas fluorescens* is a gram-negative bacterium, represented by a single-celled rod with a curved or straight long axis, one or more polar flagella, no spore formation, sheath, or stem motility (Kumar et al., 2019). *P. fluorescens* survives well at ambient temperature, and can easily contaminate refrigerated food which affects the flavor and quality of the food and is a serious threat to the food industry (Kumar et al., 2019). In addition, *P. fluorescens* are associated with some human diseases, such as septicemia, septic shock, and intravascular coagulation (Quintieri et al., 2019; Shu et al., 2019). An investigation in Italy reported that ready-to-eat vegetables were contaminated with *P. fluorescens* (Caldera and Franzetti, 2013). Another example is pasteurized milk that for a short time can still be contaminated with *P. fluorescens*, which produces luciferin that seriously affects the flavor and quality of the milk (Reichler et al., 2018).

Extensive research has been done on the antibacterial activity of linalool (Jin et al., 2020; Liu et al., 2020), but to the best of the authors’ knowledge, the mechanism underlying the antibacterial activity of linalool against *P. fluorescens* remains poorly understood. Therefore, the present study aimed to characterize the antibacterial properties of linalool and to investigate the mechanism of its antibacterial action against *P. fluorescens.* The antibacterial mechanism of linalool was investigated from two aspects: (1) The effects of linalool on the cell morphology, which mainly include the membrane potential, and intracellular macromolecular substances. (2) The effects of linalool on the intracellular metabolic activities of the bacterium which mainly includes measuring the tricarboxylic acid cycle, the glycolysis pathway, and the activities of enzymes related to respiration.

## Materials and Methods

### Materials and Chemicals

A strain of *P. fluorescens* (ATCC13525) was purchased from the Guangdong Microbial Culture Preservation Center (Guangdong, China), and incubated in a nutrient broth (30°C, 150 rpm) for 24 h to obtain the log-phase of the bacteria. Linalool was purchased from the Hainan Camphora Biotech Co., Ltd. (Hainan, China). Rhodamine 123, resazurin, and iodonitrotetrazolium chloride (INT) were purchased from Shanghai Yuanye Bio-Technology Co., Ltd. (Shanghai, China). The bicinchoninic acid (BCA), adenosine triphosphatase (ATPase) A070-2-2, malic acid dehydrogenase (MDH) A021-2-1, succinic acid dehydrogenase (SDH) A022-1-1, and pyruvate kinase (PK) A076-1-1 assay kits were purchased from Nanjing JianCheng Bioengineering Institute (Nanjing, China). Unless otherwise specified, all other chemicals were of an analytical grade.

### The Antibacterial Activity of Linalool

#### Determination of the Minimal Inhibitory Concentrations (MIC), and Minimum Bactericidal Concentration (MBC) of Linalool

The MIC and MBC of linalool were determined using the agar dilution method (Shu et al., 2019). Briefly, linalool was mixed with ethanol (10% final concentration of ethanol), and then 2 mL of this solution was added to 18 mL of solid medium to obtain antibacterial solutions with final concentrations of 0.3125, 0.625, 1.25, 2.5, and 5 μl/mL, respectively. Next, a prepared plate was coated with 200 μL of the activated bacterial suspension, and the plate was incubated at 30°C for 24 h. A sample with sterilized water was used as the blank control, and ethanol (1%) was added to a suspension of the bacterial for the negative control. The experiment was repeat three times for each concentration gradient. The MIC value was the lowest concentration of linalool which visibly inhibited the growth of the bacteria. The MBC value was the lowest concentration of linalool at which no colony was visible on the surface after 48 h of continuous culture.

#### The Growth Curve of *P. fluorescens*

The antibacterial activity of linalool was reflected in the growth curve of *P. fluorescens* (Zeng et al., 2011; Zhang et al., 2017). Activated *P. fluorescens* was diluted to 1 × 10^6–7^ CFU/mL, and linalool was diluted with 1% ethanol and added to the medium to make the final concentration 1/2MIC and 1MIC. One millilitre of the bacterial suspension was mixed with 100 mL of the prepared medium and all cultures were incubated at 30°C for 24 h. Three parallel samples were taken from each group every 2 h and the OD 600 nm value was measured with an ultraviolet spectrophotometer. The OD 600 nm absorbance value was used to measure the concentration of the bacteria in the solution and reflected the growth rate of the bacteria.

### Scanning Electron Microscopy (SEM) Observation

To determine the efficacy of the essential oil and the morphological changes of *P. fluorescens* strains, SEM observation was used on the tested bacteria (Shu et al., 2019). The *P. fluorescens* log-phase was centrifuged and washed twice with 0.1 M phosphate buffer solution (PBS, pH 7.4), then 1/2 MIC and 1 MIC of linalool were added to the resuspensions, respectively. Control group was added with the same amount of sterile water and ethanol (final concentration 1%). Next the suspensions were incubated at 30°C for 4 and 8 h, respectively, and then the suspensions were centrifuged. After this, the cells were dehydrated using sequential exposure per ethanol concentrations ranging from 30 to 100%. The dehydrated samples were pre-freezed at –20°C for 2 h and freeze-dried for 12 h. The samples were then sputter-coated with gold under vacuum and examined by SEM (S-4800, Hitachi, Tokyo, Japan).

### The Antibacterial Mechanism of Linalool Against *P. fluorescens*

A log-phase bacterial suspension was prepared and treated with various concentrations of linalool (sterile water, 1% ethanol, 1/2 MIC and 1 MIC). All cultures were incubated at 30°C and 150 rpm. For the subsequent experiments three parallel samples were collected at different time intervals.

#### Determination of the Membrane Potential (MP)

The influence of linalool on the membrane potential was evaluated using the method described by Zhang et al. (2016, 2017) and Fang et al. (2019). The prepared bacterial suspension was centrifuged (6,000 rpm, 10 min) every 30 min, washed twice with PBS and resuspended. After incubation under dark conditions for 30 min, after which rhodamine 123 with a final concentration of 2 μg/mL was thoroughly mixed into the bacterial suspension. The bacteria suspension was then washed and resuspended with PBS. Then, the fluorescence value was immediately measured at the excitation and emission wavelengths of 480 and 530 nm, respectively.

#### Loss of 260 nm-Absorbing Materials

After 0, 30, 60, 90, and 120 min of treatment, the bacterial suspension was centrifuged at 4,000 rpm, and the absorbance of the obtained supernatant was determined at 260 nm using an ultraviolet spectrophotometer (Yang et al., 2015; Diao et al., 2018).

#### Leakage of Protein From *P. fluorescens*

The leakage of protein from the bacterial cells was tested using the Bicinchoninic acid (BCA) Protein Assay Kit (Jiancheng Bioengineering Institute, Jiangsu, China). After incubation for 0, 0.5, 1, 1.5, and 2 h, the prepared bacterial were centrifuged (6,000 rpm, 10 min) and washed three times with PBS. Subsequently, prepared bacteria cells were further resuspended in PBS for ultrasonic processing (power 300 W, interval 1.1 s, 10 min) in an ice bath. Finally, the prepared homogenate was used to determine the concentration of protein leaked from the calls using the BCA Protein Assay Kit (Cui et al., 2018; Lin et al., 2018; Hu et al., 2019).

### Effect of Linalool on the Respiratory Metabolism of the Cells

#### Metabolic Activity

The metabolic activity of *P. fluorescens* with different treatments was measured using resazurin (Zhu et al., 2019). Every 30 min, 100 μg/mL of resazurin solution was added to the log-phase of the bacterial suspensions treated with different concentrations of linalool (sterile water, 1% ethanol, 1/2MIC, and 1MIC). These solutions were placed in a shaker for 2 h in the dark. Then, the samples were centrifuged at 10,000 rpm for 4 min, and the supernatant was collected. Finally, the mean fluorescence intensity (MFI) was determined using a fluorescent microplate reader at excitation and emission wavelengths of 560 and 590 nm, respectively.

#### The Activity of Respiratory Chain Dehydrogenase

Respiratory chain dehydrogenase converts the colorless iodonitrotetrazolium chloride (INT) to water-insoluble, dark red iodonitrotetrazolium formazan (INF). Subsequently, the activity of the respiratory chain was evaluated using a reducing spectrophotometric value of INF (Gomaa, 2017; Kang et al., 2018). Bacterial cultures at the logarithmic growth stage were centrifuged at 4,000 r/min for 10 min, washed and suspended with sterile normal saline. The bacterial suspension with different treatments was centrifugated (4,000 rpm, 10 min), washed three times and then resuspended with sterile normal saline. Subsequently, the 0.01 mol/L INT was prepared with methanol and water (1:1). 1,350 μL of the bacterial suspension was added to the 150 μL INT solutions to produce final concentrations of INT in solution of 1 mmol/L. The solution was placed at 30°C for 30 min and measured at 630 nm using an ultraviolet spectrophotometer.

#### The Activity of Adenosine Triphosphatase (ATPase), Malic Acid Dehydrogenase (MDH), Succinic Acid Dehydrogenase (SDH), and Pyruvate Kinase (PK)

Different concentrations of linalool (sterile water, 1% ethanol, 1/2MIC, and 1MIC) were added to the bacteria cell solutions (approximately 10^7^ CFU/mL). All samples were incubated at 30°C and 150 rpm, then centrifuged and washed three times with normal saline. Subsequently, the prepared bacteria cells were resuspended in normal saline for the ultrasonic processing (power 300 W, interval 1.1 s, 10 min) in an ice bath. The ATPase, SDH, MDH, and PK activities of *P. fluorescens* exposed to different concentrations of linalool (sterile water, 1% ethanol, 1/2 MIC, and 1 MIC) were determined using detection kits (Jiancheng Bioengineering Institute, Jiangsu, China) that were used in accordance with the manufacturer’s instructions.

### Statistical Analysis

All experiments were performed in triplicate. Data were expressed as the mean ± *SD* (*n* = 3). One-way ANOVA and Duncan’s multiple range tests were used to express the significance of differences (*P* < 0.05) between the means.

## Results

### The Antibacterial Activity of Linalool

**FIGURE 1:**
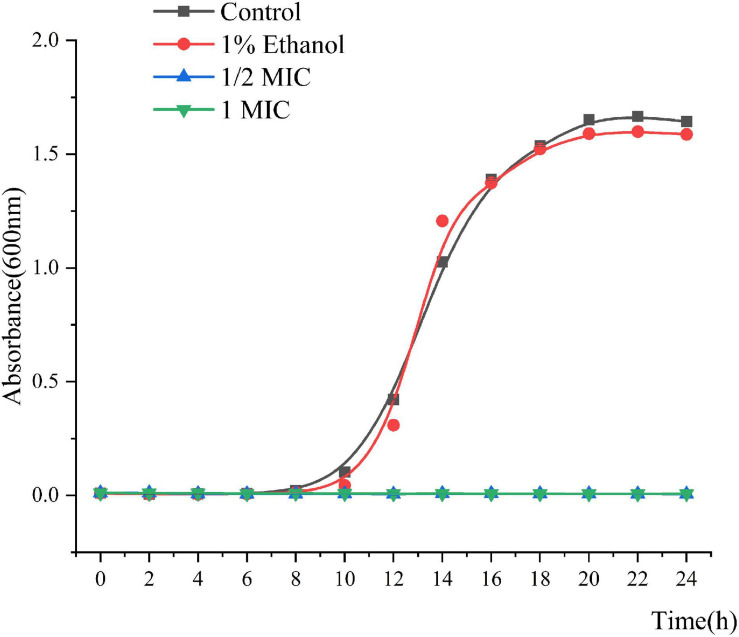
Growth curves of *P. fluorescens* ATCC 13525 treated with different concentrations of linalool. Each value indicated the average of three independent determinations.

The MIC and MBC of Linalool against *P. fluorescens* was 1.25 and 2.5 μL/mL, respectively. As shown in Figure 1, the growth curves of *P. fluorescens* generally followed the model S-shaped growth curve, and reached the logarithmic phase after 6 h and the stable stage after 18 h. However, the growth of *P. fluorescens* treated with linalool was completely inhibited, indicating that linalool had a good inhibitory effect on *P. fluorescens*.

### The Mechanism by Which Linalool Inhibits *P. fluorescens*

#### Scanning Electron Microscope (SEM)

**FIGURE 2:**
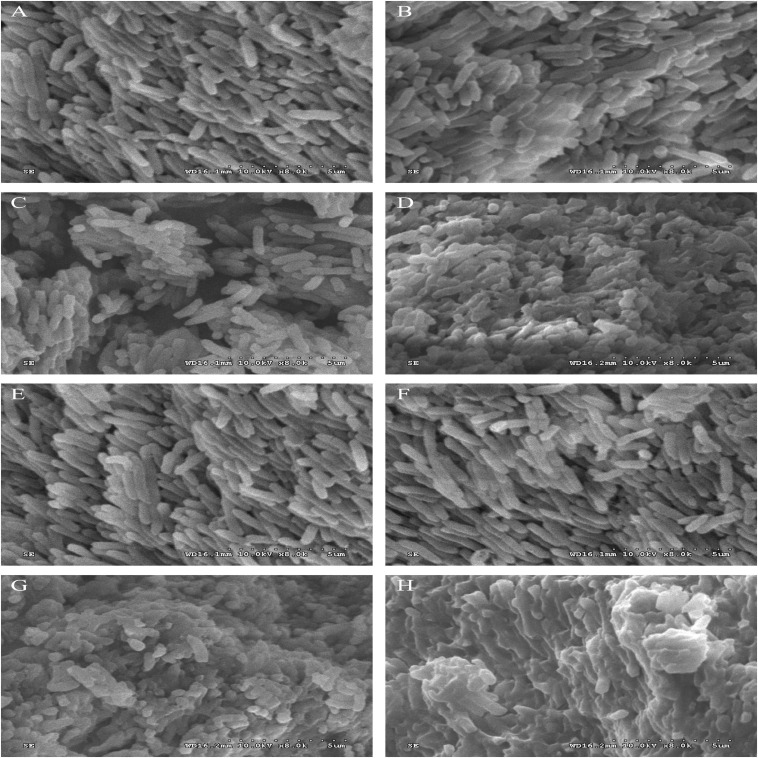
Scanning electron microphotographs of *P. fluorescens*. *P. fluorescens* untreated for 2 h **(A)**, untreated for 4 h **(E)**, treated with 1% ethanol for 2 h **(B)**, treated with 1% ethanol for 4 h **(F)**, treated with linalool at 1/2 MIC for 2 h **(C)**, treated with linalool at 1/2 MIC for 4 h **(G)**, treated with linalool at 1 MIC for 2 h **(D)**, treated with linalool at 1 MIC for 4 h **(H)**.

The morphological and physical changes of *P. fluorescens* were treated with 1/2MIC and 1 MIC for 2 and 4 h. Figure 2 shows the SEM images of the bacteria in the treated group and the bacteria in the untreated group. These images directly illustrate the damage of linalool to the bacteria. Compared with the untreated control group and 1% ethanol group, the number of abnormal strains in the processing group increased significantly. Unprocessed cells are bar, rule and complete (Figures 2A,B), while some bacteria that have been processed by linalool have been deformed, wrinkled, glued together, and part of the cell is broken (Figure 2). In addition, 2 and 1 h of images showed that the damage was obviously increased in the higher concentration of linalool and processing time.

#### Effect of Linalool on the Membrane Potential (MP)

**FIGURE 3:**
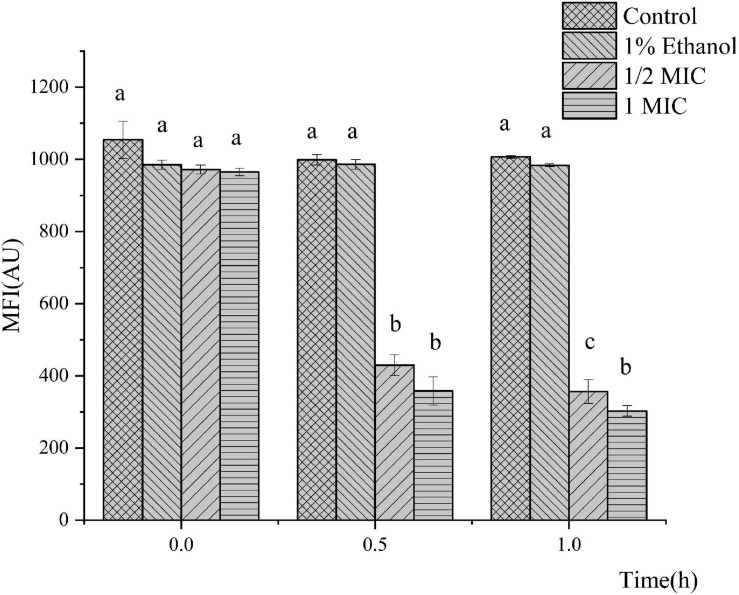
Effect of treatment with linalool on the membrane potential (MP) of *P. fluorescens*. All measurements were expressed as the mean ± *SE* (*n* = 3). Means with different letters were significantly different in MP (*P* < 0.05).

The MP was selected as an indicator to evaluate the integrity of the membranes of the cells because it plays an important role in bacterial physiology. The results of the bacterial MP are shown in Figure 3. Compared with the control, *P. fluorescens* treated with linalool in the 1/2 MIC and 1 MIC groups showed significant cell membrane depolarization because their fluorescent intensity decreased by 63.33 and 68.64%, respectively. The fluorescence intensity of *P. fluorescens* was significantly inversely correlated with the concentration of linalool (*P* < 0.05). This indicated that linalool caused significant depolarization of the cytoplasmic membrane of *P. fluorescens*.

#### Effect of Linalool on the Nucleic Acids

**FIGURE 4:**
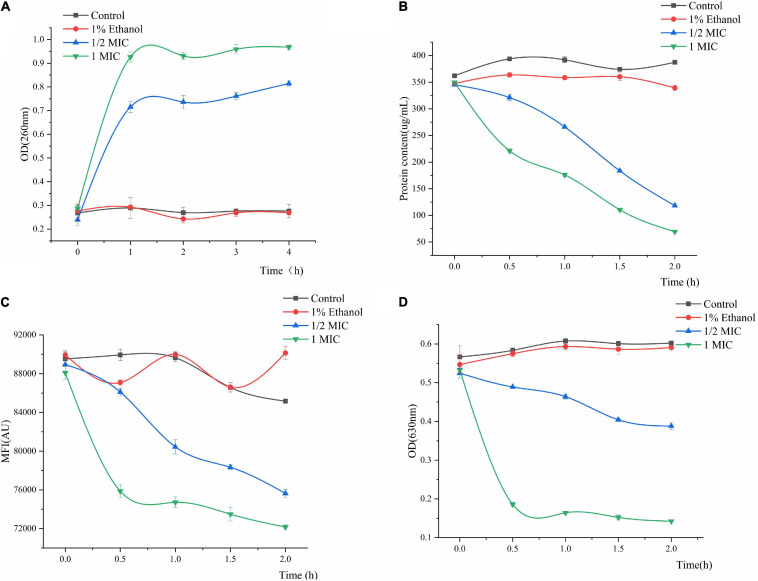
Effect of treatment with different concentration of linalool on nucleic acid **(A)**, intracellular protein **(B)**, metabolic activity **(C)**, and respiratory chain dehydrogenase **(D)** of *P. fluorescens*. Each value indicated the average of three independent determinations.

The results of *P. fluorescens* treated with linalool for 4 h (Figure 4A) indicated that the release of the cell constituents were significantly positively correlated with an increase in the concentration of linalool. Compared with the control group, after incubation with 1/2 MIC and 1 MIC linalool for 4 h, the levels of nucleic acids (OD 260 nm) increased by 2.89 times and 3.46 times (*P* < 0.05), respectively. In addition, the leakage of the intracellular nucleic acids increased significantly with an increase in the concentration of linalool (*P* < 0.05).

#### Effect of Linalool on Protein

Changes in the intracellular protein content can be observed in Figure 4B. Proteins, as important biological macromolecules in cells, usually do not leak out of the cells. Consequently, any change in the intracellular protein content of cells can be used as an indicator of the structural integrity of the cells. In Figure 4B, the protein concentration of 1MIC linalool treated bacteria was 69.42 μg/mL at 2 h. Compared with a protein content of 387.39 μg/mL for the control group, the protein content after treatment with linalool had decreased by 81.66%. Moreover, this effect of treating linalool *P. fluorescens* with linalool was more significant positively correlated with the concentration of linalool (*P* < 0.05).

#### Effect of Linalool on Metabolic Activity

The effect of linalool on the metabolic activity of *P. fluorescens* is shown in Figure 4C. The metabolic activity of the treatment group decreased with an increase in the treatment time, and decreased by approximately 11.18 and 15.27% at 2 h, respectively. The mean fluorescence intensity (MFI) of the control group fluctuated slightly, but in general, the metabolic activity of *P. fluorescens* treated with linalool was significantly inhibited (*P* < 0.05).

#### Effect of Linalool on Respiratory Chain Dehydrogenase

Regarding the activity of respiratory chain dehydrogenase, the control group only slight growth compared to the initial data, whereas the 1 MIC and 1/2 MIC groups had sharp declining trend, respectively (Figure 4D). Compared with the control group, the respiratory chain dehydrogenase activity of 1/2MIC and 1MIC groups were decreased by 35.58 and 76.37%, respectively. The activity of respiratory chain dehydrogenase was significantly inversely correlated with concentration of linalool (*P* < 0.05).

#### Effect of Linalool on Adenosine Triphosphatase (ATPase), Malic Acid Dehydrogenase (MDH), Succinic Acid Dehydrogenase (SDH), and Pyruvate Kinase (PK)

**FIGURE 5:**
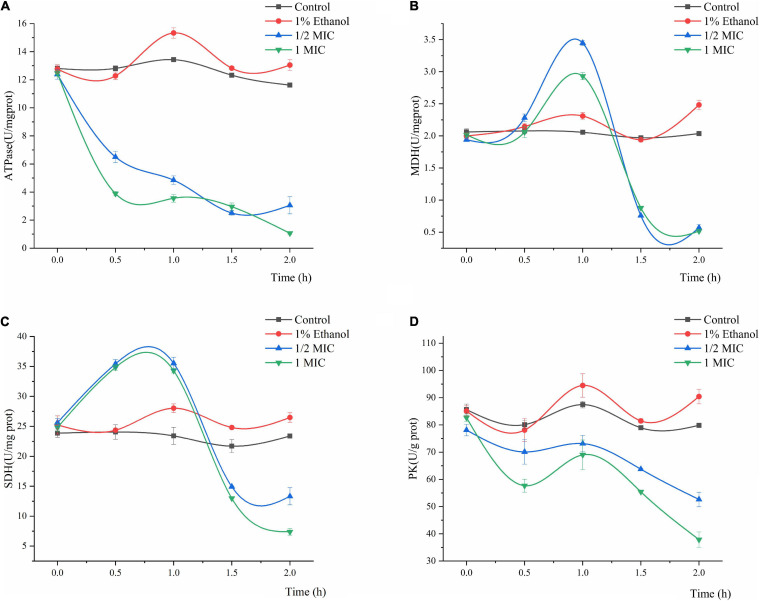
Effect of treatment with different concentration of linalool on the activity of Adenosine triphosphatase (ATPase), Malic acid dehydrogenase (MDH), Succinic acid dehydrogenase (SDH), and Pyruvate kinase (PK). **(A)** ATPase of *P. fluorescens*, **(B)** MDH of *P. fluorescens*, **(C)** SDH of *P. fluorescens*, **(D)** PK of *P. fluorescens*. Each value indicated the average of three independent determinations.

The effects of Linalool on the ATPase, MDH, SDH, and PK activities of *P. fluorescens* are shown in Figure 5. In general, the ATPase, SDH, MDH, and PK activities of *P. fluorescens* showed some natural variation, whereas the values of the control group were significantly higher than those of the treatment group (*P* < 0.05). The ATPase activity of *P. fluorescens* treated with linalool decreased sharply within the first 2 h, and the ATPase activity of the 1MIC group decreased to its minimum value of 1.0646 U/mg prot within 2 h (Figure 5A). The MDH activity of *P. fluorescens* in the two treatment groups increased in the first 0–1 h and reached maximum values of 3.4465 and 2.9314 U/mg prot, respectively and then decreased in the following1–2 h and reached minimum values of 0.5683 and 0.5162 U/mg prot, respectively (Figure 5B). Moreover, compared with the control group, the final MDH activity in the treatment group was significantly inhibited (*P* < 0.05). As shown in Figure 5C, the SDH activity of the control group varied across a narrow range, while the SDH activity in this group treated with linalool showed an increasing trend in 0–1 h, and then decreased to minimum value of 13.3136 and 7.3933 U/mg prot, respectively. The PK activity of the bacteria treated with linalool decreased significantly (Figure 5D) (*P* < 0.05). Compared with the control groups, after incubation with linalool for 2 h the PK activity of *P. fluorescens* was decreased by 34.12 and 52.57% in the 1/2 MIC and 1 MIC.

## Discussion

EOs have been widely studied in the food preservative industry because they are natural and safe (Calo et al., 2015). Linalool, as the main component of many essential oils, has been proved to have potential antibacterial activity. Some studies have shown that the EOs of *Cinnamomum camphora* (contain 69.94% linalool) (Wu et al., 2019), *Gnaphlium affine* (contain 10.62% linalool) (Zeng et al., 2011) and green huajiao (contain 28.2% linalool) (Diao et al., 2013) have a variety of antibacterial activities. The synergistic action of linalool and EOs can increase the antibacterial effect of EOs extracted from plants (Herman et al., 2016). Many authors have mentioned the antibacterial activity and antibacterial mechanism of essential oils, but the mechanism of action of plant EOs is very complicated due to its numerous components. In the present study, linalool, one of the main components of many plant EOs, was selected as an antibacterial agent to study its mechanism of action on *P. fluorescens*.

The results from the measurement of MIC and MBC, followed by the growth curve indicated that linalool had strong and consistent inhibiting effects on *P. fluorescens* (Figure 1). EOs (from *Litsea cubeba leaf, Salvia mirzayanii, Zataria multiflora, Gnaphlium affine, Forsythia koreana*) affect the structure of the cell envelope because it was found that the major antibacterial compositions of the EOs could penetrate through the cell wall and destroy the cytoplasmic membrane (Zeng et al., 2011; Kavoosi and Rabiei, 2015; Yang et al., 2015; Zomorodian et al., 2017; Nguyen et al., 2018). Scanning electron microscope (SEM) results showed that the cell morphology changed after linalool treatment (Figure 2). As a component of proton dynamics, MP is the potential difference between the inside and outside of biological cells, and it is closely related to the production of ATP. When the external environment of the cell changes, the ion concentration on opposite sides of the cell membrane will change, and the cell membrane will depolarize, resulting in a change of membrane potential and thus affecting cell metabolism (Zhang et al., 2017; Fang et al., 2019). Therefore, the mechanism of antibacterial action was reflected by the determination of MP. In the present study, we measured the membrane potential of *P. fluorescens* exposed to linalool at 1/2MIC and MIC levels (Figure 3). The results clearly showed that the treatment with linalool could lead to cell depolarization and affect the production of ATP and reduce cellular metabolic activity.

Intracellular macromolecules were selected as another aspect to elucidate the mechanism of the antimicrobial action of linalool, because the integrity of the plasma membrane is a key factor in bacterial growth (Zhang et al., 2016). In normal cells, nucleic acids and proteins are macromolecules that present in the cell membrane and cytoplasm of bacteria. Nucleic acids carry unique genetic information and participate in translation, transcription, and DNA replication, while proteins provide major structural functions. Hu et al. (2019) found that treatment with *Litsea cubeba* EO (β-Linalool 1.201%) affected the integrity of the cell membrane of *Staphylococcus aureus*, which lead to the leakage of intracellular macromolecules which included nucleic acids and proteins. Our results showed that the intracellular leakage of macromolecules increased with the addition of linalool (Figures 4A,B). Therefore, linalool may act on the cell membrane and affect the integrity of the membrane, which caused nucleic acids and proteins to be released through the fault membrane, and resulted in increased intracellular leakage of macromolecules (Diao et al., 2014).

Metabolic respiration is considered to be the main energy producing process for the germination and growth of most microorganisms. Similarly, the respiratory system is one of the most common targets for antimicrobial agents (Yang et al., 2016). Resazurin is a type of weak fluorescent substance, which can be degraded by various oxidoreductases enzymes in cells into high fluorescence resorufin. Therefore, the metabolic activity of *P. fluorescens* can be characterized by the determination of the average fluorescence intensity after treatment with linalool (Zhu et al., 2019). The results showed that the average fluorescence intensity of *P. fluorescens* decreased after treatment with linalool, indicating that its metabolic capacity was weakened (Figure 4C). INT can be converted into water-insoluble and dark red iodonitrotetrazolium formazan (INF) by the bacterial respiratory chain dehydrogenase. INT has an absorption peak at 630 nm, thus the loss of respiratory chain enzymatic activity can be assessed by the decreasing spectrophotometric value of INF (Sun et al., 2018). The results showed that the activity of respiratory chain dehydrogenase was inhibited after treatment with linalool, which was consistent with the results of the metabolic activity (Figure 4D). Bacteriostatic agents may break through the barrier of the extracellular membrane, peptidoglycan and periplasmic, and destroy respiratory chain dehydrogenase, thus inhibiting cellular respiration. The process has previously similar been reported for various antibacterial agents (Gomaa, 2017).

Adenosine triphosphate (ATP) is closely related to energy metabolism, cell function and life activities (Bajpai et al., 2013). ATPase is one of the most important enzymes to promote the production and metabolism of ATP. Its main mode of action is to provide cofactor and energy for cells, catalyze ATP to ADP, and thus provide energy for cells (Pei et al., 2019). The decline in ATPase activity may block the metabolism of carbohydrates, which can hamper cell growth and even lead to cell death (Lan et al., 2019). The decrease in the level of ATPase in *P. fluorescens* treated with linalool indicated that the activity of ATPase was destroyed by Linalool (Figure 5A). This phenomenon may lead to a decrease in the content of bacterial ATP, thus affecting the metabolic activity of the bacteria, which corresponds to the results of the MP analysis. Lin et al. (2018) also found that the bacteriostatic agent ε-PL could inhibit the respiration of *Listeria monocytogenes* by reducing the activity of ATPase.

**FIGURE 6:**
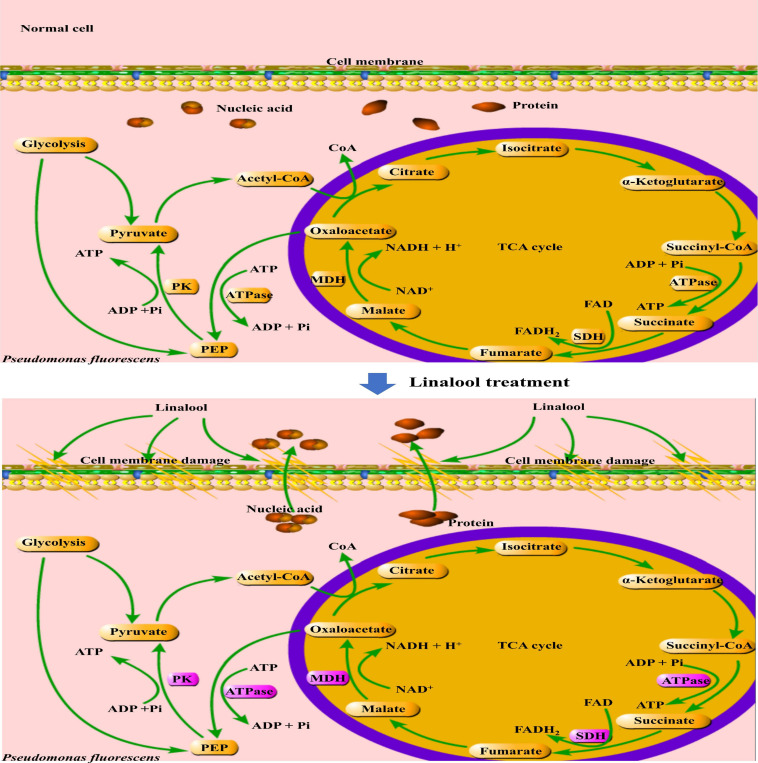
Mechanisms of stress response in *P. fluorescens*. The purple frame represents the enzyme with significantly changed activity.

In addition, the glycolysis pathway is closely related to the formation of ATP. Pyruvate kinase (PK) turns phosphoenolpyruvate and ADP, into ATP and pyruvate, respectively, and is one of the main rate-limiting enzymes in glycolysis (Wang et al., 2020). As shown in Figure 5, linalool treatment inhibited PK activity and prevented the generation of a pyruvate and ATP, thus affecting the metabolic activity of the bacteria. Under the action of pyruvate dehydrogenase system, the pyruvate undergoes oxidative decarboxylation to form acetyl-CoA. Acetyl-CoA reacts with oxaloacetic acid to form citric acid, which opens the tricarboxylic acid cycle (TCA). The TCA cycle, coupled with the energy in the mitochondria and interactions with a variety of anabolic pathways, is an essential metabolic pathway in all aerobic organisms (Fernie et al., 2004). As shown in Figure 6, SDH and MDH are both key enzymes involved in the TCA pathway in both eukaryotic and prokaryotic cells, and play a crucial role in energy metabolism in calls. SDH located on mitochondrial membrane, is the only multi-subunit enzyme involved in oxidative phosphorylation in the cell membrane. Succinate is formed from the conversion of succinyl-CoA, and then under the SDH catalytic succinate conversion to fumaric acid and simultaneously produces FADH_2_ (Akram, 2014). SDH links the respiratory metabolism and oxidative phosphorylation, therefore it is representative of the mitochondrial enzymes (Pei et al., 2019). Due to its important role in respiratory metabolism, SDH has become the target of some bacteriostatic agents. It was shown that SDH mutation was found in 14 pathogenic fungal resistant strains (Sierotzki and Scalliet, 2013). SDH activity of *P. fluorescens* after treatment with linalool indicated that bacterial respiration was weakened, which might lead to bacterial dysfunction (Figure 5C). MDH, another key enzyme in TCA, is widely found in mitochondria and bacterial cell membranes. It performs important metabolic functions in the aerobic energy production pathway, and is also involved in malic acid shuttling (Yao et al., 2011; Yang et al., 2016). MDH catalyzes L-malic acid to oxaloacetate and produces NADH (Pei et al., 2019). In the present study, treatment with linalool significantly reduced MDH activity (Figure 5B), suggesting that inhibition of the TCA cycle by linalool may result in an imbalance between aspartic acid malate shuttling and reduction equivalent.

Linalool has a strong and stable inhibitory effect on *P. fluorescens*. Treatment with linalool changed the morphology and structure of bacteria, destroyed the permeability of their cell membranes, induced the loss of intracellular macromolecules (include nucleic acids and proteins), inhibited the cells respiration rate, and reduced the activity of respiration related enzymes, including MDH, SDH, PK, and ATPase. These results suggest that linalool may inhibit metabolic respiration and disrupt enzyme activity related to energy production in *P. fluorescens*. As a result, cell membranes are destroyed, and cell metabolism is impaired.

## Conclusion

With the attention of consumers on food safety, natural and safe EOs have become a popular subject of research in the field of food preservation. In the present study, the antibacterial activity and mechanism of linalool on *P. fluorescens* was explored. The results showed that linalool had a significant inhibitory effect on *P. fluorescens*. Linalool first acts on the cell membrane, causing damage to the structure and function of the cell membrane and causing the leakage of intracellular macromolecules. In addition, linalool seemed to have an effect on the activity of certain intracellular enzymes by inhibiting enzyme activity associated with the TCA cycle and glycolysis pathway. In addition, linalool also inhibited ATPase and respiratory chain dehydrogenase, thereby inhibiting ATP production and cellular respiration. In short, linalool acts in many of the above mentioned pathways, resulting in abnormal cellular structure and metabolic function, which results in cell death. Thus, linalool is a candidate as an effective natural food preservative and multifunctional food additive. We will continue this research and further explore the antibacterial mechanism of EOs to ensure the safe and effective application of natural antibacterial agents in the food industry.

## Data Availability Statement

The original contributions presented in the study are included in the article/supplementary material, further inquiries can be directed to the correponding author/s.

## Author Contributions

FG: writing—original draft. WXC and WJC: methodology. QC and QL: data curation. MZ: software. HC and YY: formal analysis. QZ: project administration. All authors contributed to the article and approved the submitted version.

## Conflict of Interest

The authors declare that the research was conducted in the absence of any commercial or financial relationships that could be construed as a potential conflict of interest.
